# The Effect of Patient-Specific Cerebral Oxygenation Monitoring on Postoperative Cognitive Function: A Multicenter Randomized Controlled Trial

**DOI:** 10.2196/resprot.4562

**Published:** 2015-12-18

**Authors:** Lucy Ellis, Gavin J Murphy, Lucy Culliford, Lucy Dreyer, Gemma Clayton, Richard Downes, Eamonn Nicholson, Serban Stoica, Barnaby C Reeves, Chris A Rogers

**Affiliations:** ^1^ Clinical Trials & Evaluation Unit University of Bristol Bristol United Kingdom; ^2^ Department of Cardiovascular Sciences University of Leicester Leicester United Kingdom; ^3^ Bristol Heart Institute University Hospitals Bristol NHS Foundation Trust Bristol United Kingdom

**Keywords:** cardiac surgery, cardiopulmonary bypass, transfusion, cognition, valve, coronary artery, asepsis, infection, randomized clinical trial, cerebral oxygenation

## Abstract

**Background:**

Indices of global tissue oxygen delivery and utilization such as mixed venous oxygen saturation, serum lactate concentration, and arterial hematocrit are commonly used to determine the adequacy of tissue oxygenation during cardiopulmonary bypass (CPB). However, these global measures may not accurately reflect regional tissue oxygenation and ischemic organ injury remains a common and serious complication of CPB. Near-infrared spectroscopy (NIRS) is a noninvasive technology that measures regional tissue oxygenation. NIRS may be used alongside global measures to optimize regional perfusion and reduce organ injury. It may also be used as an indicator of the need for red blood cell transfusion in the presence of anemia and tissue hypoxia. However, the clinical benefits of using NIRS remain unclear and there is a lack of high-quality evidence demonstrating its efficacy and cost effectiveness.

**Objective:**

The aim of the patient-specific cerebral oxygenation monitoring as part of an algorithm to reduce transfusion during heart valve surgery (PASPORT) trial is to determine whether the addition of NIRS to CPB management algorithms can prevent cognitive decline, postoperative organ injury, unnecessary transfusion, and reduce health care costs.

**Methods:**

Adults aged 16 years or older undergoing valve or combined coronary artery bypass graft and valve surgery at one of three UK cardiac centers (Bristol, Hull, or Leicester) are randomly allocated in a 1:1 ratio to either a standard algorithm for optimizing tissue oxygenation during CPB that includes a fixed transfusion threshold, or a patient-specific algorithm that incorporates cerebral NIRS monitoring and a restrictive red blood cell transfusion threshold. Allocation concealment, Internet-based randomization stratified by operation type and recruiting center, and blinding of patients, ICU and ward care staff, and outcome assessors reduce the risk of bias. The primary outcomes are cognitive function 3 months after surgery and infectious complications during the first 3 months after surgery. Secondary outcomes include measures of inflammation, organ injury, and volumes of blood transfused. The cost effectiveness of the NIRS-based algorithm is described in terms of a cost-effectiveness acceptability curve. The trial tests the superiority of the patient-specific algorithm versus standard care. A sample size of 200 patients was chosen to detect a small to moderate target difference with 80% power and 5% significance (two tailed).

**Results:**

Over 4 years, 208 patients have been successfully randomized and have been followed up for a 3-month period. Results are to be reported in 2015.

**Conclusions:**

This study provides high-quality evidence, both valid and widely applicable, to determine whether the use of NIRS monitoring as part of a patient-specific management algorithm improves clinical outcomes and is cost effective.

**Trial Registration:**

International Standard Randomized Controlled Trial Number (ISRCTN): 23557269; http://www.isrctn.com/ISRCTN23557269 (Archived by Webcite at http://www.webcitation.org/6buyrbj64)

## Introduction

### The Clinical Problem

The development of cardiopulmonary bypass (CPB) represents one of the greatest achievements of modern medicine. By maintaining adequate perfusion through an extracorporeal pump and oxygenator, CPB enables complicated surgical procedures to be performed on the heart. Since its development in the 1950s, refinement of CPB technology has made cardiac surgery safer and widened its availability to increasingly elderly and sicker patients. Perfusion technology remains imperfect, however, and CPB is still associated with significant morbidity [1]. The pathophysiology of CPB-associated morbidity is multifactorial but includes regional hypoperfusion and tissue hypoxia, often within vascular beds, which are already abnormal due to advanced age or comorbidities such as diabetes. Consequently, up to 25% of patients may experience neurocognitive decline [2] and up to 25% experience acute kidney injury [3].

### Oxygen Delivery During CPB

Adequate tissue oxygen delivery during CPB is achieved through the optimization of several parameters including CPB pump flows, perfusion pressure, hematocrit, and the oxygen saturation of arterial blood. In contemporary clinical practice in adult cardiac surgery, the adequacy of perfusion is determined by the following: (1) the use of global measures of oxygen utilization such as the mixed venous oxygen saturation (SvO_2_), or (2) evidence of tissue hypoxia as implied by elevated serum lactate, a marker of anaerobic cell metabolism, or other indicators of metabolic acidosis. However, in some patients, particularly those with pre-existing end organ dysfunction, global measures may not detect regional hypoxia [4-7].

Direct measures of regional tissue oxygen levels such as gastric tonometry, laser Doppler flowmetry of the intestinal mucosa, or cerebral venous oxygen saturation using jugular bulb catheters can be used to measure and optimize tissue oxygenation. However, these modalities are invasive which limits their use. More recently, near-infrared spectroscopy (NIRS) has emerged as a noninvasive and accurate technique to monitor regional tissue oxygenation [8-12]. An added benefit is that NIRS can accurately measure tissue oxygenation in the brain, one of the most important end organs. NIRS sensors, when applied to the forehead, can determine the relative saturation/desaturation of blood within cerebral arterioles and venules. NIRS cerebral oximetry has been clinically validated as a measure of cerebral venous oxygen saturation [11,13,14], and is now approved as a noninvasive measure of regional cerebral oxygenation by the Medicines and Healthcare Products Regulatory Agency (MHRA). Several observational and a small randomized trial in adult cardiac surgery [8-10,12] have shown that optimization of cerebral oxygenation using NIRS can be associated not only with reduced neurological morbidity but also with a reduction in renal complications and other major adverse clinical events. The wider benefit from optimization of cerebral oxygenation observed in these studies may have arisen because the brain is more susceptible to hypoxia than other organs [4,5,7].

Whereas cerebral oximetry is widely used in pediatric cardiac surgery, the relatively low level of clinical evidence supporting its use in adult cardiac surgery has led to wide variation in its adoption, particularly as it carries additional disposable costs of up to £200 per patient. There is thus a need for high-quality evidence to address the benefits and costs of NIRS cerebral oximetry in adult cardiac surgery patients.

### Indications for Red Blood Cell Transfusion During Cardiac Surgery

Cerebral oximetry may also be used during CPB to develop goal-directed, patient-specific indicators of the need for red blood cell (RBC) transfusion if used as part of a wider algorithm designed to optimize cerebral oxygenation. The primary goal of RBC transfusion is to optimize tissue oxygenation. Currently, most RBC transfusions are given solely as a response to a hematocrit that has fallen below an arbitrary threshold and not as a response to incipient tissue hypoxia [15-17]. However, the hematocrit is a poor indicator of regional tissue hypoxia, and therefore is inadequate as an indicator of the need for transfusion.

The hematocrit below which oxygen delivery to tissues is reduced such that anaerobic metabolism occurs is known as the “critical hematocrit (Hct).” In healthy human adults very low hematocrits of less than 15 can cause organ hypoxia [5,7], and transfusion in such cases is known to be beneficial [18,19]. However, the Hct for patients during cardiac surgery is unclear; it is thought to be higher than 15, as most patients are elderly and have comorbid conditions, but it is also likely that the Hct varies considerably both between cardiac patients and individual patients over the course of the perioperative period [20].

Hct is increased by conditions which impair autoregulation such as diabetes or increased age [21,22], and during CPB. Hct is also affected by multiple factors that affect the balance of oxygen supply and demand such as hypothermia [23], rewarming [24], pump flow [25,26], or perfusion pressure [25,27]. Uncertainty about when an individual may benefit from a transfusion is reflected by the wide range in reported hematocrit transfusion thresholds used during CPB (from 17 to 25) [28-30], as well as the wide variation in transfusion rates in cardiac surgical patients across units in the UK (35-75%) [31]. Because the Hct for any particular patient at any given time is unknown, the ability to directly measure tissue oxygenation and incipient tissue hypoxia may have distinct advantages over the use of generic and prespecified hematocrit transfusion thresholds.

### Risks Associated With Red Blood Cell Transfusion

Arbitrary, generic transfusion thresholds can result in patients being undertransfused and overtransfused. In cardiac surgery, unnecessary RBC transfusion has been shown in observational studies to be associated with increased rates of stroke, renal dysfunction, infection, prolonged ventilation times, and death [32-34], although this was not confirmed in a recently reported TITRe2 randomized trial [35]. Irrespective of the effects of transfusion on postoperative morbidity, preventing unnecessary blood transfusion would reduce health care costs. Our goal is to develop transfusion algorithms that are objective, patient specific, and directed at optimizing tissue oxygenation and the adequacy of oxygen delivery. We propose that RBC transfusion should be more appropriately given in response to an objective measure of regional tissue oxygenation, as one of a group of interventions, rather than as a response to a generic, prespecified hematocrit threshold in isolation.

### Cerebral Oximetry

Existing protocols to optimize tissue oxygenation during CPB deploy manipulations of CPB (according to levels of blood markers such as lactate) and the transfusion of RBC (according to the hematocrit). These methods are used independently of each other and in response to generic thresholds, despite the fact that both have the underlying aim of improving tissue oxygenation.

In this trial we compare alternative algorithms for optimizing tissue oxygenation during CPB (ie, a currently used generic algorithm versus a patient-specific algorithm). The patient-specific algorithm differs from the generic algorithm in two key ways. First, it considers the option to give an RBC transfusion as one element of several linked interventions to optimize regional tissue oxygenation, rather than in response to a predefined hematocrit threshold in isolation. Second, it is “goal-directed” such that the algorithm is specifically targeted to maintain cerebral oxygen delivery during CPB (monitored by NIRS).

The patient-specific algorithm aims to optimize the cerebral oxygen supply and demand balance during CPB by (1) increasing oxygen supply using hyperoxygenation, increased pump flow, perfusion pressure, or hypercapnic cerebral vasodilation; (2) increasing oxygen offloading by the use of nitrates; or (3) reducing oxygen demand by deepening anesthesia [10]. Cerebral oxygen saturations approaching a low threshold in the presence of anemia (a hematocrit of 18-23) and despite optimization of other parameters suggest that the cerebral Hct is about to be reached and transfusion is indicated. Therefore, this algorithm is patient and time specific, and goal-directed to optimize a validated objective measure of tissue oxygenation. This could potentially reduce health care costs associated with unnecessary allogeneic RBC transfusions and complications associated with tissue hypoxia during CPB.

###  Aims and Objectives

We aim to compare generic and patient-specific algorithms for optimizing tissue oxygenation during CPB in adult cardiac surgery patients in a randomized controlled trial (RCT). Compared to the generic algorithm (including a hematocrit transfusion threshold of 23), we hypothesize that the patient-specific, goal-directed algorithm (based on optimizing regional cerebral oxygen saturation), combined with a prespecified “restrictive” hematocrit transfusion threshold of 18, will result in fewer RBC transfusions and will reduce complications arising from low oxygen levels during CPB. The specific objectives of this multicentre RCT are to (1) compare the effects of the patient-specific, goal-directed algorithm versus the generic algorithm in terms of cognitive function; (2) compare the effects of the patient-specific, goal-directed algorithm versus the generic algorithm with respect to a range of secondary outcomes; and (3) estimate the cost effectiveness of the patient-specific, goal-directed algorithm versus the generic algorithm and describe this in terms of a cost-effectiveness acceptability curve.

## Methods

This is a multicenter, parallel group RCT of two different algorithms for optimizing tissue oxygenation in adult cardiac surgical patients undergoing nonemergency valve surgery, with or without coronary artery bypass graft (CABG), and using CPB at mild hypothermia (32-35°C). The trial is restricted to these patient groups because they have a higher chance of needing transfusion and needing more RBC units transfused than patients undergoing isolated CABG (Trial Registration Number [ISRCTN]: 23557269).

### Trial Population and Recruitment Procedure

The target population is adult cardiac surgical patients undergoing nonemergency valve or combined CABG and valve surgery using CPB. Patients may enter the trial if they are adults (≥16 years) of either sex undergoing valve or combined CABG and valve surgery at the Bristol Royal Infirmary in Bristol, Glenfield Hospital in Leicester, or Castle Hill Hospital in Hull, give informed consent, and are suitable for allocation to either transfusion protocol. Patients are excluded from the trial if any of the exclusion criteria apply (Textbox 1).

**Exclusion criteria for the trial.**

- Patients undergoing emergency cardiac surgery.
- Patients who are prevented from having blood and blood products according to a system of beliefs.
- Patients who may have higher perioperative hemoglobin requirements or critical limb ischemia.
- Patients with congenital or acquired RBC, platelet, or clotting factor disorders.
- Patients with a neurological disorder.
- Patients with a diagnosed psychiatric disorder, drug, or alcohol addiction.
- Patients with an already identified cognitive impairment as defined by psychometric assessment or a preoperative Mini Mental State Examination score of <24 [36].
- Patients who have previously sustained a stroke, intracerebral hemorrhage, or acquired brain injury.
- Patients with a pre-existing inflammatory state.
- Patients with end-stage renal failure or patients who have undergone renal transplantation.
- Patients unable to complete the cognitive assessments required for the trial (eg, due to language difficulties, visual, or hearing impairment).
- Patients who are unable to give full informed consent for the study.
- Patients already participating in another clinical (interventional) study.

Potential trial participants are identified from surgical theatre lists and sent study information by mail or fax. They are approached on admission for surgery and after written consent has been obtained and the patient has scored 24 or greater on the Mini Mental State Examination (MMSE), he/she is randomized by a member of the research team prior to surgery. Confirmation of a participant’s identity and eligibility has to be entered into a database before the randomized allocation is generated. Some consented patients are found to be ineligible because they have a MMSE score of less than 24. Details of all patients approached for the trial and reason(s) for nonparticipation (eg, reason for being ineligible, patient or clinician preference, or patient refusal) are documented.

### Randomization

Participants are randomized in blocks of varying size in a 1:1 ratio, stratified by center and planned surgery (valve only or combined CABG and valve). Allocations are generated by computer in advance of starting recruitment and are concealed using an Internet-based system. To maintain blinding, a member of the staff who is not involved in data collection, neurocognitive assessment, or providing health care randomizes participants shortly before surgery. The allocation is given to the responsible clinical perfusionist in a sealed envelope by the trial coordinator. If surgery is unexpectedly rescheduled, participants retain their study numbers and allocation. Eligible patients who consent to participate and who have an MMSE score of 24 or greater are randomly allocated to one of two different algorithms for optimizing tissue oxygenation during CPB (Textbox 2).

**Algorithms for optimizing tissue oxygenation.**

- “Generic algorithm” (including a standard transfusion threshold): Based on global measures of oxygen utilization and includes a predefined intraoperative hematocrit transfusion threshold of 23 (current standard practice, the existing protocol).
- “Patient-specific algorithm” (including a restrictive transfusion threshold): Patient-specific, goal-directed algorithm based on the monitoring and optimization of regional cerebral oxygen saturation, combined with a predefined restrictive intraoperative hematocrit transfusion threshold of 18.

For patients allocated to the patient-specific algorithm, optimization of cerebral oxygenation uses a protocol based on that suggested by Murkin and colleagues [8]. Target regional oxygen saturation values are specified as 70% or more of preinduction values and an absolute value of 50% or more. However, if the regional oxygen saturation remains at or above these target values, but the hematocrit falls to 18, then RBC transfusion is indicated (this represents a measure to increase patient safety and protect participants from possible risks associated with the experimental intervention). Several measures are taken to maintain these targets (Table 1).

**Table 1:** Cerebral oxygenation optimization protocol to maintain cerebral oximetry readings within 70% of baseline and greater than 50%.

Measure	Description
Equipment check	Bypass pump, oximeter sensors, head and aortic cannula position
Cerebral perfusion pressure^a^ (CPP) <60 mmHg	Raise CPP > 60 mmHg by administering 0.5 mg metaraminol
If no effect and CPP < 80 mmHg	Raise CPP > 80 mmHg by administering 0.5 mg metaraminol
Partial pressure of CO_2_ in arterial blood (PaCO_2_) < 35 mmHg	Raise PaCO_2_ to 40-45 and reduce gas flows
Inspired oxygen concentration (FiO_2_) < 0.6%	Raise FiO_2_
If no effect and FiO_2_ < 1.0	Raise FiO_2_ to 1.0
Decrease cerebral metabolic rate	Increase depth of anesthesia by increasing the propofol infusion rate
Increase pump flow to maximum tolerated by venous reservoir	
Target not met and critical hematocrit is 18-23	Transfuse 1 unit of RBC

^a^CPP=mean arterial pressure (MAP)-central venous pressure (CVP).

###  Clinical Management Protocols

The following protocols are followed for all patients unless otherwise specified.

#### Surgery and Anesthesia

Details of anesthesia, operation type, complexity, and postoperative management are recorded. To avoid confounding by variables known to affect regional perfusion (eg, anesthetic depth, pCO_2_, crystalloid infusions) anesthetic management will adhere strictly to existing protocols [37], except where required according to the individual, goal-directed protocol for patients randomized to the patient-specific algorithm (Table 1).

#### Cardiopulmonary Bypass

All patients are managed according to a standard CPB protocol for valve surgery using mild hypothermic CPB and intermittent antegrade/retrograde cold blood cardioplegic arrest as previously described [37]. All distal anastomoses are conducted during a single period of aortic cross clamp. CPB pump flows, hematocrit, and arterial and mixed venous oxygen saturation are recorded every 20 minutes according to standard practice. In addition, cerebral oximetry is continuously measured in all patients for the duration of the operative procedure (see the following section).

#### Near-Infrared Spectroscopy Monitoring of Cerebral Oxygenation

Regional oxygen saturation of blood in the cerebral cortex is measured using the INVOS 5100 regional oximeter (Somanetics, Troy, MI, USA) [10]. NIRS readings are recorded for patients in both study arms for comparison. Bilateral NIRS sensors are attached to the patient’s forehead prior to preoxygenation and anesthetic induction to calculate baseline values. Cerebral oximetry is continuously measured and recorded for the duration of the operative procedure and discontinued once the patient has left the operating theater.

For patients randomized to the patient-specific algorithm (intervention group), the protocol for perioperative optimization of cerebral oxygenation/transfusion (described in Table 1) is performed. The list of interventions used to optimize cerebral oxygenation is logged. For patients randomized to the generic algorithm (control group), NIRS measurements are recorded for comparison but the clinical team is blinded to these readings in theater and optimization of tissue oxygenation is based on global measures of oxygen utilization only (ie, current standard procedure). For patients in both groups, on return to the cardiac intensive care unit (CICU), the clinical team caring for the patient is blinded to the allocation and the NIRS readings from the operative period.

#### Transfusion Protocols

RBC transfusion protocols for patients randomized to the patient-specific algorithm and generic algorithm are as described above. One unit of RBC should be transfused and the cerebral oxygenation and hematocrit levels are checked before transfusing another unit.

Clinicians are allowed to transfuse, or refuse to transfuse, in contravention of the allocated transfusion protocol but must document the reason(s) why on the study case report form (CRF). The time and volume of the transfusion administered (if applicable) will be recorded for both groups. Non-RBC blood products will be transfused using existing unit protocols [38].

### Primary Outcome

The patient-specific algorithm is designed to both prevent regional (cerebral) hypoxia and reduce the likelihood of a patient having an unnecessary RBC transfusion, compared to the generic algorithm. The primary outcome, cognitive function measured at 3 months after surgery, was chosen to measure the hypothesized benefits of preventing regional (cerebral) tissue hypoxia.

Cognitive function is assessed by a qualified examiner blinded to treatment allocation preoperatively, between 4 and 7 days postoperatively, and again at 3 months. The recommended tests [39] are performed in a fixed order (Textbox 3). In addition, to help interpret the cognitive function data, assessments related to the cognitive testing are carried out for all participants (Textbox 4).

**Cognitive domain tests and order.**

- Attention (first trial of the Rey Auditory Verbal Learning Test [RAVLT], and sustained and divided attention; Trail-Making Test parts A and B [40,41])
- Verbal memory (RAVLT [40,42])
- Visuospatial (block design from the Wechsler Adult Intelligence Scale-Revised (WAIS-R) test [43])
- Psychomotor speed (digit symbol test from the Wechsler Adult Intelligence Scale-Revised (WAIS-R) test [43])
- Executive function/verbal fluency (Controlled Oral Word Association Test [COWAT] [44])
- Motor coordination (Grooved Pegboard Test, dominant and nondominant hand [40])

**Assessments related to the cognitive testing.**

- The Wechsler Test of Adult Reading provides a preoperative measure of intellectual ability [45].
- Documentation of medications known to interfere with neuropsychological functions (including hypnotics, sedatives, neuroleptics, anxiolytics, antidepressants, and β-blockers) preoperatively, 4-7 days postoperatively, and 3 months postoperatively.
- Assessment of patient’s current mental health using the General Health Questionnaire (GHQ-30) and Hospital Anxiety and Depression Scale (HAD) [46] preoperatively, 4-7 days postoperatively, and 3 months postoperatively, to take into account the potential interaction between postoperative cognition and mood.

### Secondary Outcomes

Several secondary outcomes are characterized from the collected data (Textbox 5). Systemic inflammatory response syndrome (SIRS) is central to the diagnosis of infective complications and is defined as greater than or equal to two of the following conditions: (1) temperature over 38^o^C or below 36^o^C, (2) heart rate greater than 90 beats/minute, (3) respiratory rate greater than 20 breaths/minute or PaCO_2_ level less than 32 mmHg, and (4) white blood cell count greater than 12,000/mm^3^ or less than 4000/mm^3^. Blood test results and temperature are classified using standard reference ranges.

**Secondary outcomes.**

- Units of RBC and other blood components transfused during the operative period and postoperative hospital stay are recorded.
- Cerebral oxygenation during the operative period: NIRS readings are recorded for both groups for comparison. Monitoring starts before preoxygenation and anesthetic induction and continues until the patient leaves the theater.
- Oxygen delivery and utilization during CPB: serial measurements of oxygen delivery and utilization are collected from the clinical perfusion record.
- EuroQol EQ-5D-3L (a generic health related quality of life instrument that measures mobility, self-care, usual-care, pain/discomfort, and anxiety/depression): assessed at baseline and at 6 weeks and 3 months after surgery.
- Length of CICU or high dependency unit (HDU) stay.
- Length of postoperative hospital stay.
- Clinical outcomes defined as infectious complications (sepsis and wound infection), stroke (validated by CT scanning), ST elevation myocardial infarction accompanied by troponin >5 ng/mL, postoperative acute kidney injury (defined as AKIN criteria stage 1, 2, or 3 [47]), and respiratory complications (ie, reintubation, ventilation >48 hours, tracheostomy, or acute respiratory distress syndrome).

- Sepsis is defined when antibiotic treatment is required for suspected infection and the presence of systemic inflammatory response syndrome (SIRS) within 24 hours prior to start of antibiotic treatment. Sepsis occurring postdischarge only contributes if the event results in admission to hospital or death; wound infection is considered in the following scenario: ASEPSIS score >20, sternum, and if applicable, leg and arm. Wounds will be assessed at least once during a participant’s hospital stay. A questionnaire will be administered at 3 months to identify wound infections arising after discharge.
- For stroke diagnosis, blinded assessment of brain imaging (CT or MRI), in association with new onset focal or generalized neurological deficit (defined as deficit in motor, sensory, or co-ordination functions) will be performed.

- Cumulative resource use, cost, and cost effectiveness.
- All-cause mortality within 30 days of surgery.
- Biochemical markers of organ injury.

The following are measured from venous blood samples taken preoperatively, on return to CICU, and 6, 24, 48, and 96 hours postoperatively: (1) S100/100B (brain), (2) troponin I or T (heart), (3) creatinine clearance derived from serum creatinine (kidney), and interleukins (systemic inflammation). The use of these markers has been described previously [48,49]. Urinary creatinine and electrolytes, urinary microalbumin, neutrophil gelatinase-associated lipocalin (NGAL), interleukin 18 (IL18), liver-type fatty acid-binding protein (LFABP), and kidney injury molecule-1 (KIM-1) [50,51] (markers of tubular and glomerular renal injury) are measured from urine collected for over a 3-hour period (1 sample taken preoperatively and 3 samples taken over the first 2 postoperative days).

### Sample Size

The cognitive outcomes are continuously scaled so target differences can be specified as “standardized differences” (0.2 is small; 0.5 is moderate; and 0.8 is large) [52]. In order to detect a small to moderate target difference with 80% power and 5% significance (two tailed), 200 patients (100 per group) are recruited. Specifically, this sample size allows the trial to detect standardized differences between groups of 0.33 (0.36 at 2.5% significance) in cognitive function (adjusting for baseline) and 0.46 in cost, while allowing for up to 12% dropout across the trial. Correlations between measures are assumed to be 0.5 between baseline and postintervention measures and 0.7 between repeated postintervention measures.

This sample size also allows a standardized difference of 0.3 to be detected for biochemical markers (adjusting for baseline and using 4 repeated measures). With respect to CICU and hospital discharge, the sample size allows a hazard ratio of at least 1.65 to be detected.

### Statistical Analysis

The primary and other continuous outcomes are analyzed by regression modeling, transforming logarithmically if required, and jointly modeling baseline values where available using mixed models for repeated measures. Serial CPB oxygen delivery and utilization are compared between the 2 groups to determine whether this is improved using NIRS. Time to CICU and hospital discharge is analyzed by Cox regression.

The findings are reported as effect sizes with 95% confidence intervals. Frequencies of complications, which are too infrequent for the trial to be able to detect differences between groups, are tabulated descriptively in accordance with guidelines for reporting RCTs [53]. Analyses are based on intention-to-treat and are adjusted for center and surgery (valve or CABG and valve). For the study to conclude that the patient-specific algorithm is superior to the generic algorithm evidence of superior cognitive function for at least one domain at the 2.5% level is required (the significance level chosen to reduce the likelihood of a type 1 error, but not to be as conservative as a Bonferroni correction).

Health economic analysis are undertaken by the Health Economics Research Centre, University of Oxford. Analyses are designed and conducted to estimate the costs and likely cost of the alternative approaches identified in this protocol. These analyses help to determine whether there are likely to be improvements in patient health outcomes (such as morbidity through complications) associated with the proposed new algorithm, and whether better use can be made of scarce National Health Service (NHS) resources.

Resource use data collection are integrated, where possible, into study documentation supplemented by clinical and laboratory observational data. The analysis calculates the average cost and outcome on a per-patient basis and from this the incremental cost-effectiveness ratios for the different blood algorithms are derived, producing an incremental cost per-complication avoided. Probabilistic sensitivity analysis are used to demonstrate the impact of the variation around the key parameters in the analysis of the baseline cost-effectiveness results. This examines the impact of changing resource use in particular to help with generalizing the study results to other UK settings. The results are expressed in terms of a cost-effectiveness acceptability curve. A single analysis is performed at the completion of the study and no interim analyses and subgroup analyses are planned.

### Ethical Approval

The South West Research Ethics Committee approved the trial protocol on June 15, 2009.

### Adverse Events

Serious and other adverse events are recorded and reported in accordance with the International Conference for Harmonisation of Good Clinical Practice (ICH GCP) guidelines and the Sponsor’s (University Hospitals Bristol NHS Foundation Trust) Research Related Adverse Event Reporting Policy. Castle Hill and Glenfield Hospital will notify the coordinating center (Clinical Trials and Evaluation Unit, Bristol) of all serious adverse events. Data on adverse events are collected from the time of surgery for the duration of the participant’s postoperative hospital stay and for the 3-month follow-up period.

### Measures to Reduce the Risk of Bias

Concealed randomization prevents selection bias. To assess protocol compliance, interventions made as part of the patient-specific algorithm are logged. NIRS cerebral saturation monitoring is also conducted on patients allocated to both the generic and patient-specific groups to document whether the intervention resulted in significant separation of mean NIRS values. The NIRS values are carefully concealed from the theater team in the generic group. Surgical, anesthetic, and perfusion staff in the theater cannot be blinded to allocation but every effort is being made to ensure that patients, CICU, and other ward care staff and outcome assessors are blinded to minimize performance and detection bias. To further minimize detection bias, outcome measures are defined on the basis of objective criteria. Data are collected for all patients during surgery to characterize compliance with the randomly allocated transfusion protocol. Analysis is on the basis of intention-to-treat.

### Dissemination

The findings are disseminated by usual academic channels (ie, presentations at international meetings), as well as by peer-reviewed publications and through patient organizations and, where available, newsletters to patients. As the study compares surgical techniques there will be no commercially exploitable findings from this study.

## Results

Patients have been successfully recruited over a 5-year period. Follow-up on all study patients was completed in April 2014. Results are to be published early 2016.

## Discussion

### Key Changes to the Protocol

There have been changes to the protocol since it was first approved in 2009. The study was designed with co-primary outcomes of cognitive function and infectious complications, to measure both the hypothesized benefits of preventing regional (cerebral) tissue hypoxia and a reduction in unnecessary red blood cell transfusion. Evidence available at that time suggested that RBC transfusion was associated with increased rates of infection. However, the TiTRe2 trial [35] found consistent rates of infection with both liberal and restrictive postoperative transfusion thresholds (92% versus 53% transfused, 25% infection rate in both groups with less than 20% due to sepsis). As a result, infective complications was removed as a co-primary outcome and added as a secondary clinical outcome. Similarly, a cumulative infection score, which we had intended to develop using data from the TITRe2 trial by supplementing data on wound infections with data describing the severity of sepsis was dropped. Instead the occurrence of an infectious complication is reported. There have also been changes to the study biomarkers. Urine levels of LFABP and KIM-1 replaced cystatin C and complement activation in blood samples as more sensitive and specific biomarkers for early detection of glomerular and tubular renal injury. Glutathione-*S*-transferases alpha and pi, novel markers of tubular and glomerular renal injury, were also added but have since been removed after preliminary laboratory test results indicated technical problems with the ELISA kits. The manufacturer was informed of these findings. The blood and urine samples collected were stored for analysis at the end of the trial. The indicated changes have not impacted the integrity of the trial.

The study was initially designed as a single-center study in Bristol. To increase recruitment rates, after 2.6 years, the study was extended to 2 further centers. At the request of the funder, the sample size was reviewed after 100 participants were recruited. The correlation between baseline and postintervention cognitive function was lower than anticipated when the study was planned (0.5 rather 0.7) and there was a higher than anticipated dropout rate early on in the trial (see the following section). To account for these differences, the target sample size was increased from 150 to 200 participants. The target effect size was unchanged.

### Protocol Compliance

#### Perfusion Protocol

Perfusionists involved in the conduct of the trial are experienced in using NIRS equipment due to its routine practice in pediatric cardiac surgery. However, this trial involves the implementation of a specific and more complex NIRS protocol in addition to the routine clinical duties. Therefore, compliance of this protocol is carefully monitored. For each participant, the trial coordinator sets up the NIRS equipment in an anesthetic room and highlights the procedures involved in the generic and patient-specific algorithms prior to handing over the treatment allocation in a sealed envelope. Training sessions and meetings are arranged throughout the conduct of the trial. In order to develop an effective relationship with the perfusionist team the same 3 coordinators are involved with the set up of the NIRS equipment.

#### Blood and Urine Sample Collection

This trial involves the collection of urine samples at 4 time points and blood samples at 6 time points. These samples are predominately taken by ward staff and analyzed by scientists who have no other involvement in the trial. To make sure all samples are taken in a timely fashion we hold regular teaching sessions with ward staff and have created a sample checklist for them to complete. Research nurses and trial coordinators visit the wards throughout the day to check that all samples are up to date. To reduce the likelihood of sample misplacement, an electronic sample log linked to the freezer where the study samples are stored was developed to indicate the exact location of each study sample within the freezer.

### Challenges to Recruitment and Retention of Participants

Since the protocol was written a few measures have been introduced to help with the recruitment and retention of trial participants.

#### Recruitment

**Figure 1:**
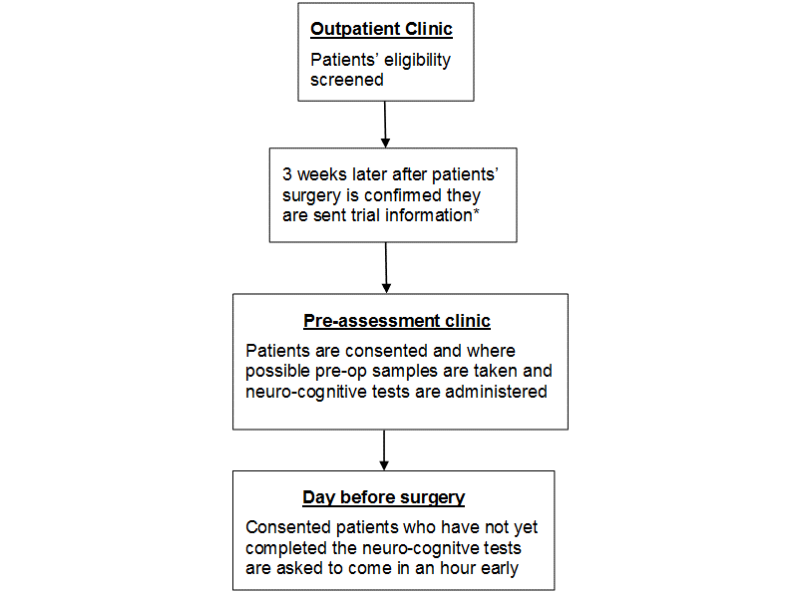
Patient recruitment pathway since February 2011.

When recruitment began, potential patients were identified from operating lists and then sent information by mail or fax. Approach and consent took place once patients arrived in the hospital for their operation. Short periods between patients receiving trial information and operation dates resulted in a number of patients refusing consent. During the course of the study, the Bristol Heart Institute introduced a day of surgery admission (DOSA) policy to maximize bed space. DOSA patients attend the hospital for a short visit the day before surgery to give consent for their procedure and undertake routine clinical tests. They then return for their surgery the following day. Around 50% of patients are now DOSA, thus limiting the amount of time to enroll patients on to the trial, conduct neurocognitive assessments, and take trial samples. To help with recruitment, ethical approval was obtained to approach and consent patients at the preassessment clinic. The pathway that most of our elective patients now follow is shown in Figure 1.

Since approaching patients earlier in their care pathway, only 3.3% (4/120) of eligible patients have refused consent due to insufficient time to consider the study compared to 11% (8/70) who previously refused on these grounds.

#### Retention

The trial requires patients to undergo neurocognitive tests at 3 time points: preoperatively, 5 days after surgery, and 3 months after surgery. These tests can be taxing and each assessment takes up to 90 minutes. As such, patients can become anxious about their performance and this may result in them refusing to complete all of the assessments. For consistency, all staff at the participating sites need to administer tests in the same way. All research team members are trained by the same chartered neuropsychologist and clinical trial co-ordinator. A core part of the training centers on an awareness of patient sensitivity. Staff are assessed and monitored throughout recruitment period.

After discharge, patients may decide they do not wish to come back to the hospital for the 3-month follow-up assessments because they may find it too stressful or may not want to travel. Therefore, where possible, we try to schedule the follow-ups with routine postoperative hospital outpatient appointments. In May 2011, ethical approval was obtained to conduct 3-month follow-up visits at patients’ homes. Since home visits have taken place, only 1.7% (2/120) of patients have refused to take part in the follow-up compared with 7% (5/70) previously. Putting these strategies in to place has helped us increase recruitment in to the trial, reduce patients lost to follow-up, and collect high-quality outcome data.

## Abbreviations

CABG: coronary artery bypass graft
CBP: cardiopulmonary bypass
CICU: cardiac intensive care unit
CVP: central venous pressure
DOSA: Day of Surgery Admission
Hct: critical hematocrit
KIM-1: kidney injury molecule-1
LFABP: liver-type fatty acid-binding protein
MMSE: Mini Mental State Examination
NHS: National Health Service
NIRS: near-infrared spectroscopy
RCT: randomized controlled trial
SvO2: mixed venous oxygen saturation

## Appendix

Multimedia Appendix 1 NHS Peer review 1.

Multimedia Appendix 2 NHS Peer review 2.
