# Supplementary material for: The Effect of Patient-Specific Cerebral Oxygenation Monitoring on Postoperative Cognitive Function: A Multicenter Randomized Controlled Trial
Source: JMIR Res Protoc. 2015 Dec 18;4(4):e137. doi: 10.2196/resprot.4562 (PMC4704972; doi:10.2196/resprot.4562)
Supplement: Multimedia Appendix 1 [file resprot_v4i4e137_app1.pdf]

NHS
National Institute for
Health Research

# Programme Grants for Applied Research	**NIHR Number:**<br>RP-PG-0407-10384
**Peer Review Form**	**Programme Title:**<br>Development of a multi-modality blood conservation strategy in cardiothoracic surgery
Please return by: 30 November 2007	
If you have any problems submitting this form please contact the CCF on:<br><br>Tel: 020 8943 7652	**Reviewer Reference Number:**<br>3

Using this Form

Thank you for agreeing to review this application to the Programme Grants for Applied Research programme. Your completed responses are considered confidential by the Programme Grants for Applied Research programme, and therefore exempt under the provisions of the Freedom of Information Act (section 41). The comments that you make in sections 1 to 7 may be fed back, unattributed, to the applicants. However, comments in sections 8 and 9 are confidential from the applicant and will not be fed back. The sections in question are clearly marked. Receipt of this document from the Programme Grants for Applied Research programme, and your subsequent completed return, form a 'mutual confidentiality agreement' covering your completed responses. This information will not be released without prior consent unless required by law.

Once you have completed your peer review please save it with the file name **FullStagePeerReviewForm3_RP-PG-0407-10384.doc**. This should be the same name as the file you downloaded. The file should then be uploaded to the CCF website. Please log on at http://www.nihr-ccf.org.uk and click on the ‘log in’ link. Once logged in click on the Programme Grants->Full Stage Peer Review link. **Only a file with the same name as that downloaded can be uploaded to the site**. This helps us track the movement of the peer review in our system.

Assessment of the Proposals

Please rate how the proposal addresses each of the selection criteria for the Programme Grants for Applied Research funding scheme in the following boxes. The prompts are intended to help you focus on the specific areas addressed by the selection criteria, but please feel free to comment on additional aspects which you consider to be relevant.

If you wish to comment only on certain elements of the proposal which fall within your area of expertise, please outline below which areas of the application you are able to review.

I am happy to comment on the proposal in general but I will concentrate particularly on the assessmentof haemostasis.

1. The strength of the research team including the relevant expertise and track-record of the applicants in conducting high-quality applied health research

- Are the applicants well qualified to undertake the proposed research, on the basis of their track record in applied health research, as judged by publication output, previous research funding, and impact on health service practice and policy?
- Does the proposed research team have the necessary breadth and depth of expertise to undertake the planned programme of research?
- Are the clinical or academic environments in which the research will be undertaken adequate for successful delivery of the proposed research programme, and are the clinical, academic, or organisational links needed to support the research, or to help translate it into practice, in place?

Cardiothoracic surgery provides a good context for this research. It does of course have coagulation and transfusion problems that are entirely its own, but the conclusions of the study should be generally applicable. The unit at Bristol have an excellent record of research in this area and should not have problems extending to the programme of work proposed here.

2. The relevance of the proposed research to the priorities and needs of the NHS

- What is the importance of the proposed research, *eg* in terms of burden of disease?
- Is there a clear need for research in this area, and a clear rationale for the proposed line of research?
- How relevant is the proposed research to the priorities and needs of the NHS?
- To what extent does the proposed work add distinct value to what is already known, or research in progress?

The premise behind this proposal and its importance to the NHS can readily be accepted. There is a very good case that we do not use transfusion (and here we refer primarily to red blood cells) to the maximum benefit of patients. In addition there is considerable uncertainty as to the most appropriate triggers for transfusion. I think that B1 in this proposal will address this well. It is also true that blood transfusion is a major expense to the NHS, primarily in the preparation and administration of blood products but also in terms of the consequent morbidity and unfortunately, mortality. This is a major issue and one of major importance to the NHS in which there is a clear need for further research.
Cardiothoracic surgery is an excellent arena in which to address this problem, which will nonetheless provide information that is likely to be readily applicable to other areas of the NHS. Thus the study has a good grounding and intends to make transfusion safer, more efficient and thereby cheaper. The principal question is whether this study is designed appropriately to achieve these aims.

B2. will this work?
I think the approaches are largely novel and will add distinct value to state of knowledge.
A. The value to be got from the validation of the bleeding prediction score seems rather less beneficial without a positive outcome from (B) which allows the risk of transfusion to be better understood. However the analysis of the relationship between inhibition of platelet function and blood loss may be useful. It will depend how much this is confounded by the effects of bypass. Will it be stratified for this?
C. What are the outcome measures? This really should be a randomised trial because of all the confounding factors.

3. The likelihood of significant benefit to the NHS and patients within a three to five year timescale

- Is there a likelihood of significant benefit to the NHS and patients within a three to five year timescale? Are potential benefits applicable to the NHS and patients generally (or just limited to the environment of the proposed programme)?
- Are the proposed plans for disseminating the results of the research, and for engaging with health care planners and policy makers, appropriate and adequate?

I think that better transfusion policies will be of widespread benefit for the NHS.The assumption is rather that we need to give less blood, but defining benefit and patients who will benefit will be a major advance. This will be applicable outside the cardiothoracic surgery setting in which this study is to be carried out.

4. The quality of the proposal

- Are the aims and objectives realistic within the timeframe and within the resources proposed and do the research plans satisfactorily address the objectives?
- Are the proposed study designs and methods for all elements of the programme appropriate, valid, robust and feasible?
- How convincing and coherent is the overall approach proposed? Is long-term, large scale programme grant funding appropriate – is there added value over and above the dividends from the individual elements?
- Have major ethical, scientific, technical or organisational challenges been identified, and will they be addressed adequately?
- Are the arrangements for the engagement of patients, their representatives, and the public in the research programme appropriate and adequate?

The individual elements of the proposal are considered below. In general I am supportive of the application which will generate a greate deal of data of great relevance to the NHS. Moreover the data have reasonably immediate applicability to an urgent problem of clincal practice in the NHS. This investment will continue to build a unit and associated groups that are likely to continue to provide valuable information after the tenure of this award. In as much as a great deal of the structures and organisation already exist, the investment is likely to prove good value. It would be counterproductive to try and perform this study in a smaller or shorter way.
The techniques applied are robust. In some respects there is an element of speculation and because of the large numbers of analyses some apparently significant findings may on repeat testing turn out to be statistical fluctuations. However, I think this is recognised in some of the modelling and in the plans of investigation.
Some elements of the study are randomised controlled trials that will require ethical approval and consent in the standard fashion but there are no fundamental ethical problems with this study.

A1. Developing and validating a transfusion risk predictor.
So far the model seems to have good sensitivity at the cost of poor specificity (24%). It is not clear that simply including more patients will improve this. Inclusion of data from other units will be useful only if they use similar transfusion protocols. Nor is it clear how the data will be used to actually reduce the frequency of blood transfusion. A ‘consensus-based blood conservation protocol’ is mentioned but not described.
Given their reasonable criticism of transfusion protocols I am surprised it is not clear that they intend to use some measure of blood loss rather than simply transfusion.

A2. Platelet function inhibition is known to affect bleeding and thrombotic risk in these patients. As the application notes, it is not known where the optimum balance of risk lies. At the same time

it has emerged that individual functional and pharmacological responses to these agents varies considerably. Therefore an assessment of platelet function may be useful. In this case the corresponding intervention is described: it may be limited to platelet transfusion but this is likely to be worthwhile. The confounding factor is likely to be the effects of prolonged CPBypass. However this is recognised and with sufficient patient numbers could be controlled for. Regrettably a limited number of tests is dictated by the large numbers. The assessment of bleeding risk will not be useful unless balanced against the risk of thrombosis which is not mentioned here. It is however included in the background on p16 so presumably the data will be available and should be included in outcome markers here.

B1. Patient-specific transfusion regimen. I think this is an attractive part of the proposal and perhaps the one with theoretically the greatest potential for benefit. I am not expert to comment on the technology involved but it has a reasonable literature to support it. The use of tissue oxygenation as an indication for transfusion seems entirely appropriate. It is only disappointing that by retaining a threshold of 18 compared to the control level of 23 the applicants have given themselves a very narrow window in which to show benefit.

B2. Previous attempts to use standard coagulation tests to predict bleeding have not been successful. There are some data to support the use of TEG guided therapy but they are limited. The proposal here attempts to improve on this. Most of the ‘specialised’ tests chosen are ones that will not be detected by conventional tests and therefore whose importance may not yet have been appreciated. Moreover, the tests will be assessed after the bypass and therefore more likely to detect the actual defect in relation to bleeding. As pointed out in the application, the variance of many of these tests in the post pump population is unknown. However it will be determined by this study and may in itself be a very useful outcome.

C. Washing rbc to reduce inflammatory response. This has a reasonable grounding and could prove to be important in this group of patients. Unlike the other aspects of the study it may be less applicable to the practice of transfusion in general even in other surgical settings. As with coagulation there is the potential for confounding by numerous other operation related factors.

5. Justification for Resources Requested

- Are the requested resources, including staffing, clearly justified? Are they essential for the work proposed?
- Taking into account the expected benefits of the work proposed and the level of resources requested, do the proposals promise good value for money?

Overall, the amount requested for a 5 year study of this size with very large number of patients and analyses looks good value. I don't have any specific criticisms of the items or salaries requested.

6. Additional comments

Do you have any further comments or suggestions on how the proposed programme of research might be improved?

None

7. Overall score

Please provide an overall score (lowest score 1, highest 6) for the application that can be used by the Programme Grants for Applied for Research Selection Panel. The scoring system is as follows:

6	Excellent	Proposed research programme acceptable as it stands
5	Good	Proposed research programme acceptable with minor changes
4	Good potential	There is much merit in this proposal, but the programme could only be considered acceptable after resubmission, perhaps with additional external support.
3	Some merits	There are significant weaknesses in this application, but these could in principle be addressed.
2	Poor	Weak application
1	Extremely poor	Unsupportable application

Please select using the drop down box below.

Please select...

8. Confidential comments

If you would like to make any comments in confidence concerning this application, please enter them in this field. In particular, please indicate whether you see any limitations as critical and, if so, whether you think that the underlying problems are potentially correctable by this research team.

9. Conflict of interest

We believe that to make the best decisions on full proposals, we should know about any competing interests that referees may have. We have already conducted our own checks to rule out any institutional (work-place) conflicts. Are you aware of any potential competing interests that you may have? If you are in any doubt about any potential competing interest then please declare it. We will not reject your opinion simply because you declare a competing interest, but we would like to know about it.

Please select...

If yes, please give details:
