# Supplementary material for: The Effect of Patient-Specific Cerebral Oxygenation Monitoring on Postoperative Cognitive Function: A Multicenter Randomized Controlled Trial
Source: JMIR Res Protoc. 2015 Dec 18;4(4):e137. doi: 10.2196/resprot.4562 (PMC4704972; doi:10.2196/resprot.4562)
Supplement: Multimedia Appendix 2 [file resprot_v4i4e137_app2.pdf]

NHS
National Institute for
Health Research

<table>
<tr><td>Programme Grants for Applied Research</td><td>NIHR Number:
RP-PG-0407-10384</td></tr>
<tr><td>Peer Review Form</td><td rowspan="3">Programme Title:
Development of a multi-modality blood conservation strategy in cardiothoracic surgery</td></tr>
<tr><td>Please return by: 30 November 2007</td></tr>
<tr><td rowspan="2">If you have any problems submitting this form please contact the CCF on:

Tel: 020 8943 7652</td></tr>
<tr><td>Reviewer Reference Number:
6</td></tr>
</table>

Using this Form

Thank you for agreeing to review this application to the Programme Grants for Applied Research programme. Your completed responses are considered confidential by the Programme Grants for Applied Research programme, and therefore exempt under the provisions of the Freedom of Information Act (section 41). The comments that you make in sections 1 to 7 may be fed back, unattributed, to the applicants. However, comments in sections 8 and 9 are confidential from the applicant and will not be fed back. The sections in question are clearly marked. Receipt of this document from the Programme Grants for Applied Research programme, and your subsequent completed return, form a 'mutual confidentiality agreement' covering your completed responses. This information will not be released without prior consent unless required by law.

Once you have completed your peer review please save it with the file name **FullStagePeerReviewForm6_RP-PG-0407-10384.doc**. This should be the same name as the file you downloaded. The file should then be uploaded to the CCF website. Please log on at http://www.nihr-ccf.org.uk and click on the ‘log in’ link. Once logged in click on the Programme Grants->Full Stage Peer Review link. **Only a file with the same name as that downloaded can be uploaded to the site**. This helps us track the movement of the peer review in our system.

Assessment of the Proposals

Please rate how the proposal addresses each of the selection criteria for the Programme Grants for Applied Research funding scheme in the following boxes. The prompts are intended to help you focus on the specific areas addressed by the selection criteria, but please feel free to comment on additional aspects which you consider to be relevant.

If you wish to comment only on certain elements of the proposal which fall within your area of expertise, please outline below which areas of the application you are able to review.

As requested I have focussed on reviewing the quality of the proposed analyses methods. I have made further comments in the other sections but please note that these comments are limited as I have little knowledge of blood tranfusions and the existing research in this area.

1. The strength of the research team including the relevant expertise and track-record of the applicants in conducting high-quality applied health research

- Are the applicants well qualified to undertake the proposed research, on the basis of their track record in applied health research, as judged by publication output, previous research funding, and impact on health service practice and policy?
- Does the proposed research team have the necessary breadth and depth of expertise to undertake the planned programme of research?
- Are the clinical or academic environments in which the research will be undertaken adequate for successful delivery of the proposed research programme, and are the clinical, academic, or organisational links needed to support the research, or to help translate it into practice, in place?

Based on the CVs of the applicants and description of the research team (Q11), the research team is very well qualified to undertake the proposed research: The team includes several highly experienced researchers who have the depth of expertise needed, with national and international reputations in their respective fields. Further, the team comprises professionals from a broad range of disciplines including clinicians, a senior epidemiologist, senior statistician, and a health economics specialist, giving the team the necessary breadth of expertise. The statistical analyses are complex in parts and it is good therefore to see that a senior statistician is a co-applicant and that there is further funding for another statistician part-time. The inclusion of the chairman and secretary of the South West Regional Transfusion Committee and the Director of Regional Haemostasis Laboratory Services in the research team, as well as providing consultancy on transfusion medicine, will aid dissemination of findings and translation of the research into practice.

Different team members, alone or in pairs, are taking the lead on the five different studies of the programme. As long as team members input on the studies that they are not leading as well as those that they are, each study will be adequately supported. For example, clinicians inputting on studies led by health researchers and vice versa. It would be good to verify that team members will be involved to some extent in studies that they are not leading.

The BHI should be an ideal base for this research, with the dedicated Clinical Trials and Evaluation Unit and the close partnership with the UBHT, which will enable clinicians to work closely with researchers, supporting the research and helping to translate it into practice. The research team comprises a member of the Health Economics Research Centre in Oxford thereby building on established links with this leading UK centre.

2. The relevance of the proposed research to the priorities and needs of the NHS

- What is the importance of the proposed research, *eg* in terms of burden of disease?
- Is there a clear need for research in this area, and a clear rationale for the proposed line of research?
- How relevant is the proposed research to the priorities and needs of the NHS?
- To what extent does the proposed work add distinct value to what is already known, or research in progress?

Whilst blood transfusions are a life-saving treatment, there is increasing awareness of the importance of not using transfusions indiscriminately in surgery because of the associated adverse effects on patients and the diminishing availability of blood supplies. Yet the fact that there is wide variation in transfusion rates across different UK centres indicates that transfusions are being used unnecessarily. There is therefore a clear need for the proposed comprehensive programme of research into establishing when transfusions should be given. This has also been identified as an NHS priority and is important not only in terms of patient care, but also in terms of the cost to the NHS of the limited resource of blood.
I am not familiar with the literature on blood transfusions so cannot comment on how much research has already been done in this area, beyond what the applicants have reported. The applicants state that there is currently a lack of high quality evidence on appropriate use of blood transfusions. The proposed research program will be a comprehensive multidisciplinary major investigation into the use of blood transfusions. Further, in terms of adding distinct value, the applicants state that the first part of the programme will envolve development of the first validated

system for predicting bleeding and transfusion risk in cardiac surgery (UK cardiac surgery transfusion risk score). In addition, the second part of the programme will be looking at the role of measurement of regional cerebral oxygenation as a transfusion indicator, which has not been examined before. The third part of the program, investigating the extent to which washing devices can attenuate the inflammatory response to allogenic tranfusions, could have an important impact on blood safety.
In summary, given the wide variation in transfusion rates across the NHS, which suggests a lack of clear guidelines on transfusion use, and the need to reduce unnecessary transfusions, I think that the proposed research could be highly valuable and relevant to the NHS.

3. The likelihood of significant benefit to the NHS and patients within a three to five year timescale

- Is there a likelihood of significant benefit to the NHS and patients within a three to five year timescale? Are potential benefits applicable to the NHS and patients generally (or just limited to the environment of the proposed programme)?
- Are the proposed plans for disseminating the results of the research, and for engaging with health care planners and policy makers, appropriate and adequate?

The applicants state clearly plans for disseminating and implementing the results for each of the five projects that make up the programme (Q10). These dissemination plans appear to be both appropriate and adequate, and generally likely to take place within 3 to 5 years. Proposed implementation plans include:-
-first project A1: dissemination of the risk score developed as a calculator on the internet by year 5.
-second project A2: revision of current guidelines on time of withdrawal of anti-platelet medication before surgery
-third project B1: routine implementation of the goal-directed tranfusion protocol under consideration (if proven effective), to be audited in year 5.
-fourth project B2: Implementation of POC coagulopathy tests to complement the TEG (although an RCT would probably be needed first, so benefit to the NHS within 5 years may be limited here).
-fifth project C: Implementation of washing of red blood cells prior to transfusion, if found to be effective, through rewriting of protocols. The applicants state that this cannot happen within the time frame of the programme but would happen subsequently.

It is clear, and the applicants emphasize, that the benefits of the proposed research would not be limited to patients within the host division of cardiac surgery, UBHT, but findings could be translated both to other NHS trusts across the UK and to other types of surgery. This is particularly apparent for project A1 which envolves validation of a risk score in several different populations including use of data from the Edinburgh cardiac unit, the North West Quality Improvement Programme and the Society for Cardiothoracic Surgery in Great Britain and Ireland National Audit.

4. The quality of the proposal

- Are the aims and objectives realistic within the timeframe and within the resources proposed and do the research plans satisfactorily address the objectives?
- Are the proposed study designs and methods for all elements of the programme appropriate, valid, robust and feasible?
- How convincing and coherent is the overall approach proposed? Is long-term, large scale programme grant funding appropriate – is there added value over and above the dividends from the individual elements?
- Have major ethical, scientific, technical or organisational challenges been identified, and will they be addressed adequately?
- Are the arrangements for the engagement of patients, their representatives, and the public in the research programme appropriate and adequate?

This program has 5 separate projects. I comment first on each project in turn:

A1 Validate the Bristol Transfusion Risk Score in a UK wide cohort

The validation analyses look sound. They include both temporal and external validation which is important in terms of generalising the findings across the NHS, beyond the host institution. The sensitivity of the risk score is excellent at over 97%. However the specificity is noticeably low at 24%. I realise that sensitivity and specificity cannot both be arbitrarily high and it is probably more important to correctly identify patients who need tranfusions than those who don't, however as the aim of the programme is to limit unecessary transfusions, the specificity does appear low to me. Can this be improved? There is no sample size calculation for this project. That said, in reference [60] it is noted that literature on calculating sample sizes in validation studies is scarce.

A2 Evaluate the role of preoperative laboratory measurement of platelet function as a predictor of bleeding and transfusion requirement

The statistical analyses for objectives 1 and 2 are described in detail and it is good to see that the analyses include control for confounders and consideration of possible departures from linearity. In terms of sample size calculations, the applicants state that a sample of size 1000 will have >95% power to detect "significant associations" between continous exposures and outcomes at a 2-tailed significance level of 0.01. However, it would be useful to know what these significant associations are to verify this sample size calculation. The example of the power calculation for a categorical outcome is correct.

This may be due to my lack of knowledge of transfusion medicine, but I did find the overall research plans for this project difficult to follow - I felt that it wasn't clear how the objectives related to the overall project aim, and the third objective was rather vague. Whilst the analyses methods for objectives 1 and 2 are described clearly, it was not clear to me what methods would be used for objective 3. Further clarification of how the objectives meet the aim and of objective 3, is needed.

B1 Evaluate the effects of a patient-specific, goal-directed, transfusion algorithm on cognitive function, infection and transfusion.

The research methods for this project are appropriate and the optimal approach, being a randomised controlled trial, with stratified randomisation and blinding of patients and outcome assessors. The statistical analyses are appropriate, with mixed models to take account of repeated measures and Cox regression to model the time to ICU and hospital discharge. Sample size calculations are based on a lower power of 80%. It would be useful to have further details of the sample size calculations. The first two estimated standardised differences appear correct; however I estimated the third to be 0.3 rather than 0.4 but it would be good to have further details to verify this. Further details would also be needed to verify the detectable hazard ratio of 1.65.

B2 Develop a directed management algorithm for intra-operative coagulopathy using improved point-of-care evaluation of haemostasis

The research methods are described and include appropriate use of ROC curves for estimating diagnostic accuracy. However, the methods for objectives 3 and 4 need to be described more clearly, in particular, the modelling procedures to be used for objective 4. The sample size considerations are desribed well and are correct - refer to reference [78]. This study will involve the same patients as for project A2 - this is a good use of resources.

C Evaluate red cell washing and re-suspension prior to transfusion as a means of reducing transfusion associated morbidity

Optimal research methods are used here: single centre randomised controlled trial with blinding for investigators and stratified randomisation. Patients are likely to be, but not definitely, blinded - however as the outcomes are measured from blood samples and not patient reported this should not introduce bias. The analysis plan and statistical methods are to be the same as for B1 - this is appropriate. The sample size calculation looks reasonable, but more details would be useful to double-check the figures.

Overall, the aims and objectives of the programme as a whole appear realistic given the timeframe and resources. Generally, the research plans are sound and satisfactorily address the objectives with the possible exception of A2 for which the analysis plans are less coherent, as discussed above.

The added value in combining these five projects in this program is in providing a needed comprehensive overview of the appropriate use of tranfusions, combining research on different aspects and using different techniques and methods. It might be useful to summarise in some way how the separate projects fit together and give an estimate of the overall impact of the projects combined.

The applicants detail the ethical approval that is needed and will be sought. The BHI Research Advisory Group, made up of members of the public who are stakeholders in the use of, or

delivery of, healthcare and healthcare research, including patients, has been involved in the development of the programme. This group will continue to be involved in the program, in the development of patient information documents, study monitoring, sterring committees, dissemination of findings etc. In my view these arrrangements for public involvement in the proposed research are appropriate and adequate.

5. Justification for Resources Requested

- Are the requested resources, including staffing, clearly justified? Are they essential for the work proposed?
- Taking into account the expected benefits of the work proposed and the level of resources requested, do the proposals promise good value for money?

The majority of the funds requested is for staffing (£1,279,844). All additional staff (senior researcher, programme manager, part-time statistician, part-time health economist, research nusres, biomedical scientist) are clearly justified and there is a definite need for each in the programme. The remainder of the costs are for consumables, office expenses, travel, two consultancies and equipment. Again all theses costs have been justified. Travel and subsistence costs appear considerable at first glance but upon looking at the breakdown of these costs they are reasonable. Three quarters of the requested funding for equipment (£29,950) is for a single instrument: a Chrono-log 700 lumiaggregometer. The applicants highlight the value and need for this equipment not only for the current programme but in future related research. Note though that there is no mention of how easy it would be to obtain data from the society of Cardiothoracic Surgery in Great Britain and Ireland for project A1 and if there are any costs involved in accessing this data, this does not appear to be included in the programme costs.

Because of my limited knowledge of this area of medicine, I do not feel that I can comment on whether the programme overall is "good value for money".

6. Additional comments

Do you have any further comments or suggestions on how the proposed programme of research might be improved?

Provided the existing evidence on the use of blood tranfusions is as limited as the authors state I feel that the proposed research, taking on board the minor comments on the research methods, could be highly valuable and relevant to the NHS.

7. Overall score

Please provide an overall score (lowest score 1, highest 6) for the application that can be used by the Programme Grants for Applied for Research Selection Panel. The scoring system is as follows:

6	Excellent	Proposed research programme acceptable as it stands
5	Good	Proposed research programme acceptable with minor changes
4	Good potential	There is much merit in this proposal, but the programme could only be considered acceptable after resubmission, perhaps with additional external support.
3	Some merits	There are significant weaknesses in this application, but these could in principle be addressed.
2	Poor	Weak application
1	Extremely poor	Unsupportable application

Please select using the drop down box below.

Please select...

8. Confidential comments

If you would like to make any comments in confidence concerning this application, please enter them in this field. In particular, please indicate whether you see any limitations as critical and, if so, whether you think that the underlying problems are potentially correctable by this research team.

9. Conflict of interest

We believe that to make the best decisions on full proposals, we should know about any competing interests that referees may have. We have already conducted our own checks to rule out any institutional (work-place) conflicts. Are you aware of any potential competing interests that you may have? If you are in any doubt about any potential competing interest then please declare it. We will not reject your opinion simply because you declare a competing interest, but we would like to know about it.

Please select...

If yes, please give details:
